# Radiographic Outcomes of Casting Versus Splinting for Conservatively Treated Metacarpal Fractures

**DOI:** 10.7759/cureus.27643

**Published:** 2022-08-03

**Authors:** Terence L Thomas, Tyler W Henry, Jacob Tulipan, Pedro Beredjiklian

**Affiliations:** 1 Orthopedic Surgery, Thomas Jefferson University, Philadelphia, USA; 2 Orthopedic Surgery, Rothman Orthopaedic Institute, Philadelphia, USA; 3 Hand Surgery, Rothman Orthopaedic Institute, Philadelphia, USA

**Keywords:** angulation, fracture, hand, radiograph, splint, cast, metacarpal fracture, hand fracture

## Abstract

Introduction

While many have studied alternate forms of casting for conservative treatment of metacarpal fracture, few have compared casting and splinting. This study aims to compare radiographic alignment in metacarpal shaft and neck fractures immobilized with splints to those treated with casts.

Methods

A retrospective review was conducted to identify all metacarpal fractures treated by a single orthopedic hand surgeon from 2016-2020. Patients with metacarpal shaft or neck fractures treated nonoperatively, immobilized with either a cast or a splint, and with a minimum of one follow-up visit were included. Degrees of radial/ulnar angulation, dorsal/volar angulation, and changes in angulation were measured. Mean angulation measurements and changes in angulation were compared across groups using Mann-Whitney U tests.

Results

A total of 61 patients, 45 treated with casts and 16 with splints, met our inclusion criteria. The average immobilization time was 28 days for both groups (p=0.958). Change in radial/ulnar angulation was similar between the two groups (splint = -3⁰, cast = -3⁰, p=0.79). No significant differences were found when comparing changes in dorsal/volar angulation across groups (splint = -0.3⁰, cast = -0.1⁰, p=0.57). No complications were reported in either group.

Conclusions

Our results suggest that metacarpal shaft and neck fractures treated with splints can maintain fracture reduction and angulation comparable to casting. Splints offer additional benefits of reduced costs with improved patient hygiene and satisfaction. Further studies on the utility and cost-effectiveness of splints for treating metacarpal fractures are warranted.

## Introduction

Metacarpal fractures are among the most common fractures of the upper extremity [1]. The majority of these fractures can be effectively treated with immobilization for three to four weeks. Proper immobilization is essential for maintaining reduction and preventing decreases in functional outcomes due to malunion [2-3]. When compared, most cast, brace, and splinting options for immobilizing metacarpal fractures produce comparable functional results [4-5]. Therefore, it is likely that existent nonoperative treatment strategies are driven by surgeon experience and preference.

Two common immobilization methods for metacarpal fractures include short-arm casting and splinting [4]. The proposed benefits of casting are rigid maintenance of reduction and thus improved functional outcomes. However, cast immobilization often requires subsequent clinical follow-up and may limit good hand hygiene [6-8]. While splinting may provide the benefits of improved hand hygiene and patient satisfaction, patient compliance remains a concern for physicians when treating fractures with removable splints [9-10]. Though studies have compared the functional and radiographic outcomes of alternate casting methods based on metacarpophalangeal/interphalangeal joint position and mobilization [11-12], there is a paucity of evidence comparing the effects of casts versus splints in maintaining radiographic alignment of metacarpal fractures [13].

The purpose of this study is to assess the radiographic alignment of metacarpal shaft and neck fractures immobilized with splints compared to those treated with casting. We hypothesize that fractures treated with splints will have similar maintenance of radiographic alignment compared to casts.

## Materials and methods

Institutional review board approval, with a waiver of informed consent, was obtained prior to the initiation of the study. A retrospective database search was conducted to identify all patients with metacarpal fractures treated by a single fellowship-trained orthopedic hand surgeon from 2016-2020. An additional review of electronic medical records and an archived digital imaging software system (PACS, Sectra Medical, Linköping, Sweden) was used to establish our study cohort. Patients who had metacarpal shaft or neck fractures and were treated nonoperatively, immobilized with either a cast or a splint, and had a minimum of one follow-up visit were included. Exclusion criteria consisted of patients with displaced fractures requiring manipulation or operative fixation, first metacarpal or metacarpal base fractures, those lost to follow-up, and/or missing initial injury or follow-up radiographs. The decision to perform a reduction or surgically fixate was based on a combination of factors, including initial displacement, angulation, comminution, and digital malrotation. The included fractures did not meet surgical criteria based on their treating physician.

The decision to cast or splint a fracture was determined based on physician preference. Following clinical evaluation by their physician, patients were treated by an orthotist where both cast and splint groups were immobilized to include the metacarpophalangeal joints in flexion and allow for full interphalangeal joint motion. Patients treated with casts remained casted until subsequent clinical follow-up unless removal or exchange was requested by the patient (e.g., the cast became wet or dirty). Patients that were splinted were instructed to remove their splints for hygiene purposes only. Treatment protocol for both groups involved follow-up in four to six weeks to assess for fracture healing, where a decision was made to either continue or discontinue their respective cast or splint. All patients included in this study discontinued their immobilization at their first four- to six-week follow-up visit.

Patient demographics were extracted and all radiographs were evaluated for radial/ulnar and dorsal/volar angulation using anteroposterior (AP) and lateral X-ray views. Demographics included patient age, sex, injured digit(s), injured hand, handedness, fracture pattern, fracture location, type of immobilization, and immobilization period. Patients were divided into two groups based on the type of immobilization received (cast or splint). Degrees of radial/ulnar and dorsal/volar angulation were retrospectively measured by a blinded reviewer using the medullary canal method [14]. Angulation measurements were evaluated from initial injury X-rays and at the first four- to six-week follow-up visit when their immobilization was discontinued. Imaging was performed following the removal of the patient's casts/splint and therefore did not bias our blinded reviewer. The change in angulation from initial visit to follow-up visit was calculated for each patient.

Descriptive statistics were used to report patient demographics, angulation measurements, and changes in angulation. Mean angulation measurements and changes in angulation were compared across groups using the Mann-Whitney U test. Statistical significance was established at p < 0.05. An a priori power analysis was performed to determine the sample size required to detect a 10⁰ difference in angulation. Prior studies have identified standard deviations of 9⁰ and 10⁰ in metacarpal fractures treated with casts [7] and splints [15], respectively. Thus, it was indicated that 16 patients in each group were needed to sufficiently power (ß = .20) the study.

## Results

A total of 61 patients, 45 treated with casts and 16 with splints, met our inclusion criteria. Patient demographics of those immobilized with either cast or splint are detailed in Table 1. The final study population included 35 males and 13 females with a mean age of 37 years (Table 1). The average immobilization time for all patients was 28 days (splint = 28 (SD=12), cast = 28 (SD=6), p=0.958). There were no significant demographic differences between those treated with splints vs cast (Table 1).

**Table 1:** Demographics of patients immobilized with splints versus casts

Variable	Splint (N=16)	Cast (N=45)	P-Value
Age (SD)	38 (25)	35 (23)	0.63
Sex			0.77
Male	13	35	
Female	3	10	
Digit			0.86
2	0	2	
3	2	5	
4	3	8	
5	11	30	
Injured Hand			0.64
Right	11	28	
Left	5	17	
Handedness			0.37
Right	10	35	
Left	2	4	
Both	1	1	
Fracture Type			0.48
Comminuted	3	9	
Oblique	7	12	
Spiral	1	9	
Transverse	5	15	
Location			0.26
Neck	9	18	
Shaft	7	27	
Immobilization Duration (Days [SD])	28 (12)	28 (6)	0.96

**Figure 1:**
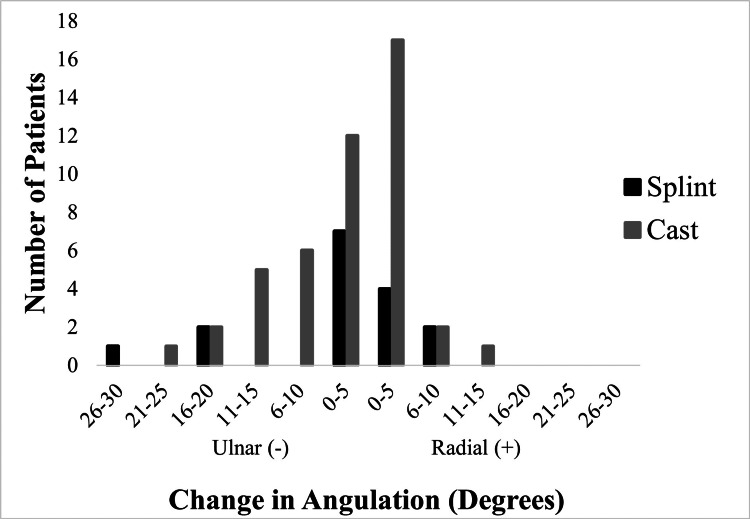
Change in radial+/ulnar- angulation following immobilization with splints versus casts

**Figure 2:**
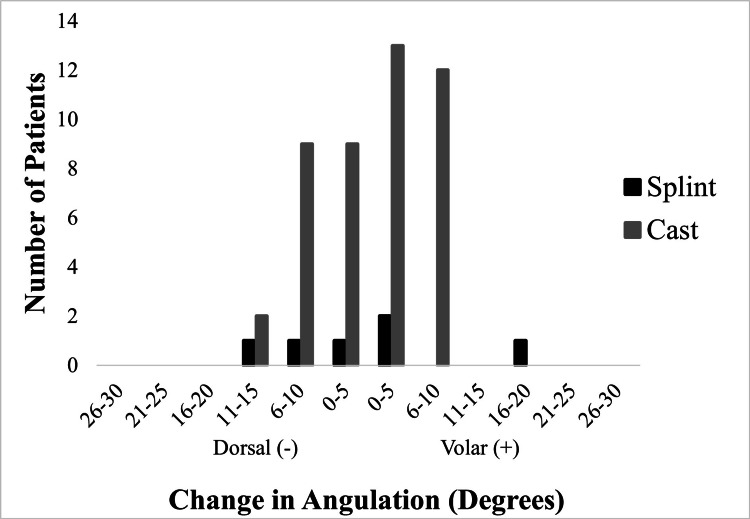
Change in volar+/dorsal- angulation following immobilization with splints versus casts

Angulation measurements and changes in angulation among patients treated with either casting or splinting are presented in Table 2. Change in radial/ulnar angulation was similar between the two groups (splint = -3⁰, cast = -3⁰, p=0.79) (Figure 1). Similarly, no significant differences were found when comparing changes in dorsal/volar angulation across groups (splint = -0.3⁰, cast = -0.1⁰, p=0.57) (Figure 2).

**Table 2:** Comparison of angulation measurements after immobilization with splints versus casts Angulation measurements are reported as mean (SD) degrees. Change in angulation calculations reported as mean (range) degrees.

Variable		Splint	Cast	P-Value
AP view (Radial+/Ulnar-)	Initial Visit	20 (16)	14 (18)	0.36
	Follow-up Visit	19 (21)	16 (20)	0.77
	Change in Angulation	-3 (-28 to 7)	-3 (-24 to 13)	0.79
Lateral view (Dorsal+/Volar-)	Initial Visit	27 (16)	25 (11)	0.97
	Follow-up Visit	27 (13)	25 (11)	0.72
	Change in Angulation	-0.3 (-11 to 17)	-0.1 (-14 to 8)	0.57

No pressure wound complications were reported in either group.

## Discussion

Metacarpal fractures are common, comprising 36% of all hand fractures [1]. Studies in conservative management of metacarpal fractures have compared alternate methods of casting and functional immobilization [4-5], but to our knowledge, few have compared casts and splints for treating metacarpal fractures [13]. We, therefore, aimed to investigate the utility of splints in treating metacarpal fractures and their ability to maintain fracture alignment comparable to those treated with casts. The results indicated that patients immobilized with splints and casts had similar changes in dorsal/volar and radial/ulnar angulation over their immobilization period. This indicates that splints and casts are equally effective at maintaining reduction and alignment following four weeks of immobilization.

Consensus is lacking regarding the optimal nonoperative treatment of metacarpal fractures. Moreover, existent studies comparing conservative management strategies are of low quality [16-18]. In a systematic review, Poolman et al. found that most conservative treatment approaches yield excellent functional outcomes, suggesting no single method is superior to the next for managing fifth metacarpal neck fractures [4]. When investigating radiographic alignment and clinical outcomes of ulnar gutter splints and plaster casts in fifth metacarpal fractures, Gulabi et al. demonstrated no superiority of either immobilization technique in terms of reduction, although splints demonstrated a lower risk of pressure wounds [13]. Our findings further reinforce this first conclusion, as patients immobilized with splints maintained reduction similarly to those treated with casts.

Reports on the benefits of splints over casts for fracture management have been primarily limited to pediatric fracture management. Removable splinting has been shown to produce comparable functional outcomes to casts while improving patient and healthcare costs and convenience [19-20]. Beals et al. studied the cost-effectiveness of Velcro ulnar gutter bracing versus casting for pediatric metacarpal neck fractures and found a bracing approach to significantly reduce both patient costs and overall healthcare burden [19]. Further studies comparing splints and casts in pediatric wrist fractures suggest that splint management provides acceptable angulation and superior function to casts while also being more cost-effective and convenient for patients and families [20-22]. Despite these reported benefits, some physicians remain resistant to splint immobilization due to concerns about patient noncompliance [10]. While these findings show promise for the potential utility of splints in metacarpal fracture care, further study is warranted that investigates clinical outcomes and cost-effectiveness of splint immobilization in skeletally mature individuals.

The present study has several limitations. First, this was a retrospective chart review where neither the physician nor the patient was blinded to the treatment approach. The decision to immobilize patients with either a splint or cast was based on surgeon preference and shared decision-making with the patient. This carries a clear risk of introducing selection bias. Second, although the study cohort was sufficiently powered to detect a difference of 10⁰ difference in angulation, we are reporting on a relatively small sample of patients, particularly in the splint treatment group. A larger prospective, randomized study is necessary to strengthen our findings. Third, we do not report on functional outcomes as a result of splint or cast immobilization. While existent literature suggests that functional outcomes are comparable across the two methods [4], we are unable to make any claims regarding functional outcomes. Fourth, variability in initial visit angulation measurements was observed across our patient cohort. This may lead some to question why many of these fractures were treated conservatively and not surgically indicated. There is currently no strong evidence to suggest that fracture angulation alone, whether in the coronal or sagittal plane, is an indication for surgical management. Rather, the assessment of digital rotation serves as a stronger metric to determine whether surgical intervention is necessary. Last, angulation measurements of fifth metacarpal neck fractures have been shown to hold notable intra-observer and interobserver variability [14]. There is likely to be an estimated 10⁰ of variability in angulation measurements among a single reader [14]. While the use of an additional blinded reviewer, in theory, may have improved the internal validity of angulation measures, existing evidence suggests that there still remains poor reliability even across multiple reviewers [14]. In an attempt to minimize bias, our independent reviewer was blinded to immobilization type during angulation measurements and used a single measurement strategy.

## Conclusions

Our findings suggest that metacarpal shaft and neck fractures treated conservatively with splints have a comparable ability to maintain fracture reduction and angulation when compared to casting. While benefits of casting are optimal alignment and better patient compliance, splints offer the proposed advantages of reduced costs and improved patient satisfaction. Further high-quality randomized studies of the utility and cost-effectiveness of splints for treating metacarpal fractures in skeletally mature patients are warranted.

## References

[1] Karl JW, Olson PR, Rosenwasser MP (2015). The epidemiology of upper extremity fractures in the United States, 2009. J Orthop Trauma.

[2] Ali A, Hamman J, Mass DP (1999). The biomechanical effects of angulated boxer's fractures. J Hand Surg Am.

[3] Birndorf MS, Daley R, Greenwald DP (1997). Metacarpal fracture angulation decreases flexor mechanical efficiency in human hands. Plast Reconstr Surg.

[4] Poolman RW, Goslings JC, Lee JB, Statius Muller M, Steller EP, Struijs PA (2005). Conservative treatment for closed fifth (small finger) metacarpal neck fractures. Cochrane Database Syst Rev.

[5] Bloom JM, Hammert WC (2014). Evidence-based medicine: metacarpal fractures. Plast Reconstr Surg.

[6] Jones AR (1995). Reduction of angulated metacarpal fractures with a custom fracture-brace. J South Orthop Assoc.

[7] van Aaken J, Fusetti C, Luchina S (2016). Fifth metacarpal neck fractures treated with soft wrap/buddy taping compared to reduction and casting: results of a prospective, multicenter, randomized trial. Arch Orthop Trauma Surg.

[8] Smith KR (2010). Boxer's fracture. Ann R Coll Surg Engl.

[9] Boutis K, Howard A, Constantine E, Cuomo A, Somji Z, Narayanan UG (2015). Evidence into practice: pediatric orthopaedic surgeon use of removable splints for common pediatric fractures. J Pediatr Orthop.

[10] Williams BA, Palumbo NE, Phillips SA, Blakemore LC (2020). What they want - caregiver and patient immobilization preferences for pediatric buckle fractures of the wrist. Iowa Orthop J.

[11] Tavassoli J, Ruland RT, Hogan CJ, Cannon DL (2005). Three cast techniques for the treatment of extra-articular metacarpal fractures. Comparison of short-term outcomes and final fracture alignments. J Bone Joint Surg Am.

[12] Hofmeister EP, Kim J, Shin AY (2008). Comparison of 2 methods of immobilization of fifth metacarpal neck fractures: a prospective randomized study. J Hand Surg Am.

[13] Gulabi D, Avci CC, Cecen GS, Bekler HI, Saglam F, Merih E (2014). A comparison of the functional and radiological results of Paris plaster cast and ulnar gutter splint in the conservative treatment of fractures of the fifth metacarpal. Eur J Orthop Surg Traumatol.

[14] Leung YL, Beredjiklian PK, Monaghan BA, Bozentka DJ (2002). Radiographic assessment of small finger metacarpal neck fractures. J Hand Surg.

[15] Kaynak G, Botanlioglu H, Caliskan M (2019). Comparison of functional metacarpal splint and ulnar gutter splint in the treatment of fifth metacarpal neck fractures: a prospective comparative study. BMC Musculoskelet Disord.

[16] Braakman M, Oderwald EE, Haentjens MH (1998). Functional taping of fractures of the 5th metacarpal results in a quicker recovery. Injury.

[17] Statius Muller MG, Poolman RW, van Hoogstraten MJ, Steller EP (2003). Immediate mobilization gives good results in boxer's fractures with volar angulation up to 70 degrees: a prospective randomized trial comparing immediate mobilization with cast immobilization. Arch Orthop Trauma Surg.

[18] Harding IJ, Parry D, Barrington RL (2001). The use of a moulded metacarpal brace versus neighbour strapping for fractures of the little finger metacarpal neck. J Hand Surg Br.

[19] Beals C, Lin J, Holstine JB, Samora JB (2019). Reducing healthcare costs in the management of pediatric metacarpal neck fractures. Pediatr Qual Saf.

[20] Hill CE, Masters JP, Perry DC (2016). A systematic review of alternative splinting versus complete plaster casts for the management of childhood buckle fractures of the wrist. J Pediatr Orthop B.

[21] von Keyserlingk C, Boutis K, Willan AR, Hopkins RB, Goeree R (2011). Cost-effectiveness analysis of cast versus splint in children with acceptably angulated wrist fractures. Int J Technol Assess Health Care.

[22] Plint AC, Perry JJ, Correll R, Gaboury I, Lawton L (2006). A randomized, controlled trial of removable splinting versus casting for wrist buckle fractures in children. Pediatrics.

